# Students’ performance of and perspective on an objective structured practical examination for the assessment of preclinical and practical skills in biomedical laboratory science students in Sweden: a 5-year longitudinal study

**DOI:** 10.3352/jeehp.2023.20.13

**Published:** 2023-04-06

**Authors:** Catharina Hultgren, Annica Lindkvist, Sophie Curbo, Maura Heverin

**Affiliations:** 1 Division of Clinical Microbiology, Department of Laboratory Medicine, ANA Futura, Karolinska Institutet, Stockholm, Sweden; 2 Division of Clinical Immunology, Department of Laboratory Medicine, ANA Futura, Karolinska Institutet, Stockholm, Sweden; 3 Division of Clinical Chemistry, Department of Laboratory Medicine, ANA Futura, Karolinska Institutet, Stockholm, Sweden; Hallym University, Korea

**Keywords:** Longitudinal studies, Students, Sweden, Undergraduate medical education

## Abstract

**Purpose:**

It aims to find students’ performance of and perspectives on an objective structured practical examination (OSPE) for assessment of laboratory and preclinical skills in biomedical laboratory science (BLS). It also aims to investigate the perception, acceptability, and usefulness of OSPE from the students’ and examiners’ point of view.

**Methods:**

This was a longitudinal study to implement an OSPE in BLS. The student group consisted of 198 BLS students enrolled in semester 4, 2015–2019 at Karolinska University Hospital Huddinge, Sweden. Fourteen teachers evaluated the performance by completing a checklist and global rating scales. A student survey questionnaire was administered to the participants to evaluate the student perspective. To assess quality, 4 independent observers were included to monitor the examiners.

**Results:**

Almost 50% of the students passed the initial OSPE. During the repeat OSPE, 73% of the students passed the OSPE. There was a statistically significant difference between the first and the second repeat OSPE (P<0.01) but not between the first and the third attempt (P=0.09). The student survey questionnaire was completed by 99 of the 198 students (50%) and only 63 students responded to the free-text questions (32%). According to these responses, some stations were perceived as more difficult, albeit they considered the assessment to be valid. The observers found the assessment protocols and examiner’s instructions assured the objectivity of the examination.

**Conclusion:**

The introduction of an OSPE in the education of biomedical laboratory scientists was a reliable, and useful examination of practical skills.

## Graphical abstract

[Fig f5-jeehp-20-13]

## Introduction

### Background/rationale

Biomedical laboratory scientists (BLS) work in different clinical laboratories (such as chemistry, microbiology, and transfusion medicine), but also within for example research laboratories and pharmaceutical companies. They perform a wide range of laboratory assays on tissue samples, blood, and body fluids which are crucial for the health sector and today approximately 60% to 70% of all diagnoses given are based on part of the analyses performed by a BLS [1]. BLS is today a licensed health profession in many countries and the core competencies includes carrying out laboratory work, analysis, and assessment [2]. The emphasis is on validation and quality assurance [3]. When practical skills are examined, the assessment is frequently unreliable and largely dependent on the examiners’ training [4]. An early innovation to improve practical evaluation is the objective structured clinical examination (OSCE) which later was extended to the practical examination, objective structured practical examination (OSPE) described in 1975 and in greater detail in 1979 by Harden and his group from Dundee [5]. It has been found to be objective, valid, and reliable. We have not found any publication where an OSPE is used as an assessment tool to evaluate BLS students’ all competencies. However, it has been used in other fields such as pharmacology and pathology [6,7]. For the assessment of BLS competencies, an OSPE can be designed to test various skills, for example, (1) general laboratory skills such as choice and handling of equipment/accessories, (2) interpretation of laboratory results, conclusions, (3) specific laboratory techniques but also (4) preclinical skills such as sampling techniques, communication, and attitude. For this purpose, an agreed checklist, instructor’s manual, and response questions are used regarding the above-mentioned aspects for the evaluation of students’ competencies. The observer evaluates the students according to a checklist and instructor’s manual provided.

### Objectives

The purpose of this study was to find students’ performance of and perspective on OSPE as an assessment tool for the examination of practical and preclinical skills in biomedical laboratory medicine for second-year BLS students. The specific research questions were to (1) study the perception, (2) acceptability, and (3) usefulness of OSPE from the students’ and examiners’ point of view through survey-questionnaire. We hypothesized that students’ passing rates would increase after participation in a previous OSPE.

## Methods

### Ethics statement

Ethical approval was not required for this study, as per the Swedish Ethical Review Authority tool. This study did not include a clinical trial and did not collect any personal data. Participation in the survey was optional for the participants, and only anonymous data were included.

### Study design

This was a 5-year retrospective longitudinal study involving biomedical laboratory science students. It is described according to the STROBE (Strengthening the Reporting of Observational Studies in Epidemiology) statement (https://www.strobe-statement.org/).

### Setting

The study was conducted over a 5-year period (spring 2015 to 2019) at Karolinska University Hospital Huddinge, Sweden. The OSPE implementation is available from Supplements 1 and 2.

### Participants

**Fig. 1.**
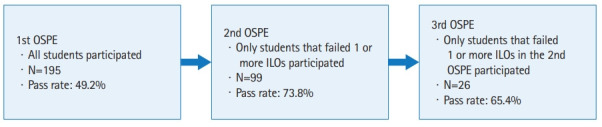
Outline of the objective structured practical examination (OSPE). The flowchart depicting outline of the OSPE including number of participants per OSPE run and pass rate. ILO, intended learning outcome.

Biomedical laboratory science students enrolled in the Biomedical laboratory science program during semester 4 at Karolinska Institutet, Stockholm, Sweden. The inclusion criteria were all students (n=198) who were supposed to take part in the OSPE. Exclusion criteria were incomplete data (e.g., students who dropped out and did not take part in the examination). A total of 195 students participated and 3 did not and these were therefore excluded. If the students passed all intended learning outcomes (ILOs), they passed the OSPE and did not take part in a rerun. Details on the implementation of the OSPE are available from Supplement 1. During the second OSPE, 96 students participated and during the third OSPE, 26 students participated (Fig. 1). The student surveys were performed after the completion of the first OSPE, but before the students were notified of the result from the first exam. A total of 99 students (50%) agreed to complete the questionnaire.

### Variables

Passing rate of OSPE is the primary outcome.

### Data sources/measurement

**Fig. 2.**
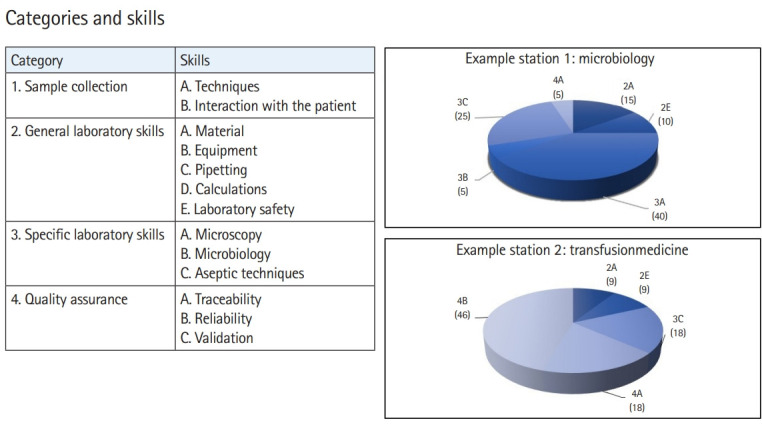
Categories and skills. The table outlines the categories and skills, while the pie charts depict what skills are assessed at 2 of the 9 stations (microbiology and transfusion medicine).

**Fig. 3.**
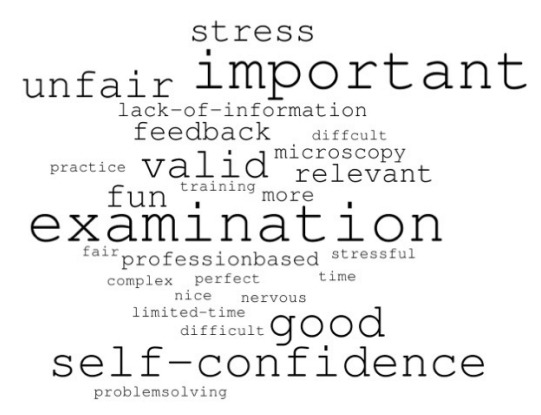
Student perceptions on the objective structured practical examination. A word cloud based on free text answers from the 63 of the 99 students who completed the student survey. Words with similar meanings but different or incorrect spellings were corrected.

Students’ performance data were generated after each OSPE (Dataset 1). Which ILOs that were assessed are depicted in Fig. 2. Furthermore, the perceptions of the examiners were evaluated both by 4 independent observers of test situations and by interviews with 5 of the participating examiners. The student perception was evaluated using anonymous voluntary survey forms with free text questions (Supplement 3). The free-text questions were further analyzed by grouping words according to resemblance and performing a word cloud (Fig. 3). Participants’ responses are available from Dataset 2.

### Bias

No notifiable bias can be detected because most target students (195/198) participated in the study. Since participation in the survey questions was voluntary, there may be some bias in this aspect.

### Study size

The sample size was not estimated since most target students were enrolled.

### Statistical methods

Data were analyzed using Excel ver. 2016 (Microsoft Corp.) and Prism 9 (version 9.5.0, Graph Pad Software). Descriptive statistics summarized the distribution and frequency of pass rates. Comparisons between groups were made by one-way analysis of variance followed by post hoc Tukey’s test. Significance was set at a=0.05.

## Results

### Participants

A total of 195 students (80,5% female) completed the OSPE examination and of these 99 also completed the student survey (response rate=50%). Table 1 displays the students’ characteristics. As examiners, 14 teachers participated and finally, 4 different teachers participated as quality observers during 2016–2019.

### Main results

#### Students’ performance of the OSPE

**Fig. 4.**
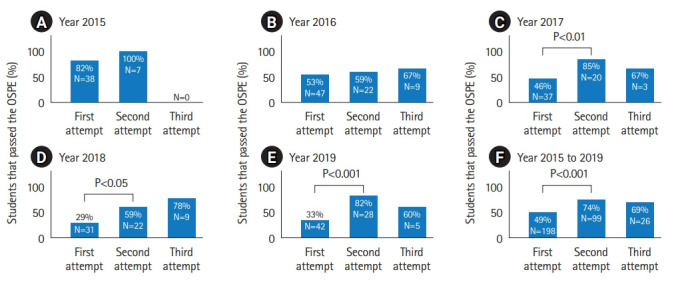
Success rate in objective structured practical examination (OSPE) divided per year. Percentage of passed biomedical laboratory science students at 3 consecutive OSPE attempts in results from 2015 (A), results from 2016 (B), results from 2017 (C), results from 2018 (D), results from 2019 (E), and finally in combination of all years 2015 to 2019 (F). Number of participants at each occasion is indicated within each bar, if statistically significant difference this indicated by the respective P-values; if not then nonsignificant.

During the first OSPE, the success rate in the OSPE varied between 29 and 81.8%, and in total 99 students failed 1 or more ILOs during the first OSPE (Fig. 4A–E). In total between 2015 and 2019, from the first OSPE run, there was a success rate of 49.2% (Fig. 4F). Among the students that participated in the second attempt, a much higher success rate was achieved (varying between 59.1% to 100%; mean 73.7%) that is 73 students passed the OSPE and only 26 failed (Fig. 4). Finally, a third OSPE run was performed (except for 2015), with the remaining 26 students and then 17 students managed to pass all ILOs and 9 failed to do so, that is a somewhat lower success rate of (varying between 60% to 77.8%; mean 65.4%) (Fig. 4). There was a statistically significant difference between the first and the second repeat OSPE (P<0.01) during 2017 to 2019, but not during the first 2 years. Nor was there any statistically significant difference between the first and the third attempt (Fig. 4). The OSPE assessed 4 different competencies (Supplement 1), sample collection, general and specific laboratory skills, and quality assurance. The highest Likert score (pass) varied between 44.1% to 95.9% for the individual stations; with a mean of 68.9% for all stations (Dataset 1).

#### Students’ perception of the OSPE

The voluntary student survey form was completed by 99 of the 198 students (50%) and only 63 students responded to the free-text questions (32%). In general, their opinions in the free-text questions were either defined as clearly positive or negative towards the OSPE, as is summarized in the word cloud in Fig. 3. These opinions ranged from positive reflections such as “fun and valid examination” and more negative reflections mentioning stress and the examination to be unfair.

#### Examiners’ perception of the OSPE

Regarding their different stations, most of the examiners agreed that the examination was useful and well-organized and that the tasks that the students were asked to perform at each station were fair, and the OSPE was a standardized examination for the assessment of preclinical and laboratory skills. Though some of the examiners’ mentioned that the OSPE might have unwanted effects on student behavior, such as just trying to “pass” the station rather than carefully carrying out the task they were asked to perform. The examiners also addressed the importance of agreed guidelines and thereby new and more specific guidelines could be included. The semi-structured interview questions are available from Supplement 4. Finally, some of the examiners stated that they would need more time for training on beforehand.

#### Quality observers’ point of view

The observers found a certain degree of subjectivity among the examiners, especially when the beforehand training was stated as insufficient by the examiners. They found that guidelines for the examiners were a key factor for objectivity of the assessment. With proper and agreed checklists they found the OSPE to a relevant assessment to discriminate between good and not so good performers and speculated that the variability that could be observed between examiners could be reduced with training.

## Discussion

### Key results

The aim was to find students’ performance and perspective on OSPE as an assessment tool for the examination of practical and preclinical skills for second-year BLS students. Almost 50% of the students passed the initial OSPE and during the repeat OSPE, 73% of the students passed. According to the students that responded to the survey some stations were perceived as more difficult, albeit they considered the assessment to be relevant. The observers found the assessment protocols and examiner’s instructions assured the objectivity of the examination.

### Interpretation

#### Students’ performance of the OSPE

There was a higher success rate during 2015/2016 (Fig. 4A, B) versus 2017, 2018, and 2019 (Fig. 4C–E). Also, the higher pass rate from the first OSPE in 2015 was statistically significant when compared to all other first OSPE occasions (with P-values <0.01, <0.5, <0.01, and <0.001, respectively). We speculate that the main reason for this is better checklists and training of the examiners. It has been shown that the assessment method influences and drives student learning [8,9]. Thus, assessing different components such as performing laboratory tests, analyzing, and interpreting laboratory data would drive students to learn these competencies. This was supported by the finding that the students performed better in the second OSPE (P<0.01). In addition, the frequency of passed examinations was higher also in the third OSPE, although this difference was not statistically significant.

#### Students’ perception of the OSPE

Most of the free-text answers reflected that they found the examination to be relevant for their future profession and in some cases, even an opportunity to practice what they have learned. The main negative opinions stated in the survey were lack of time, stress, and the examination being unfair. Some mentioned the stations being easy versus others being hard. A notion that aligns with the fact that the Likert rating also varied between the different stations, whether this has an impact on the summative result of the OSPE remains to be clarified. Though, since only 50% of the participants did respond to the survey and only 32% gave free-text answers these do not mirror the reflections of all students, possibly more of those that were either very content or discontented with the OSPE. These feelings were though uninfluenced by their exam results since the survey was performed before the students were notified of their exam results.

#### Examiners’ perceptaion of the OSPE

In general, the examiners had a positive perception of the usefulness of the OSPE, but also stated some comments that were important to address such as the need for beforehand training and agreed checklists.

#### Quality observers’ point of view

There is some evidence suggesting that examiner training and the use of different examiners for different stations reduce examiner variation in scoring and individual assessor bias [10]. To reduce examiner variation and assessor bias different examiners and some rotation between stations were applied. This also highlighted the importance of agreed checklists, instructor’s manuals, and premade response questions and the notion from examiners for the need for training beforehand.

### Comparison with previous studies

This study agrees with the objective structured laboratory examination (OSLE) that has been previously used to assess practical competencies achieved by students in a lab-based program (undergraduate biomedical science program) in Malaysia and was there perceived to be useful both by students and by faculty though the OSLE is similar to the OSPE, in the OSPE also preclinical skills such as sampling of blood (including communication) is included [11]. The notion that the student performed better during their second attempt in the OSPE is in line with previous studies stating that there is a strong positive correlation between students’ performance on in-training examinations and their final OSCE result [12]. It is also in concordance with previous literature supporting the use of OSPE as a well-accepted assessment tool for practical competencies according to both students and examiners [13].

### Limitations and generalizability

There are limitations in the analysis of the student perspective in this study, such as the fact that only 32% of the students provided reflections in free-text form, and thereby these data do not represent the perspectives of all students. Most of the students that did answer the free text form either expressed rather strong negative or positive attitudes. The study used a global rating system and is therefore also limited in its generalizability. Considering the results of this study, the OSPE can be applied in BLS to assess both preclinical and laboratory competencies and these results may apply to BLS students in other institutes in Sweden.

### Suggestions

Further research should investigate whether a different scaling system influences the outcome, and more easily can differ between a borderline failure and a borderline pass. To determine how the cut score changes when the scale changes. In this setting, there were 4 ILOs covered. It is also necessary to study how the cut score changes depending on the content and combination of the stations [14].

Since one of the major drawbacks in the students’ perception was stress and other measures to address this issue should be taken. One possible way to reduce stress and make them more familiar with the OSPE would be to participate in a peer-led OSPE [15].

### Conclusion

This study demonstrates that the OSPE provides an opportunity to test a student’s ability to integrate knowledge and preclinical and practical skills that are a must for any student aspiring to become a successful BLS. From this study, it can be concluded that the introduction of an OSPE in the education in BLS was a practical and useful examination of practical competencies.

## Figures and Tables

**Figure f5-jeehp-20-13:**
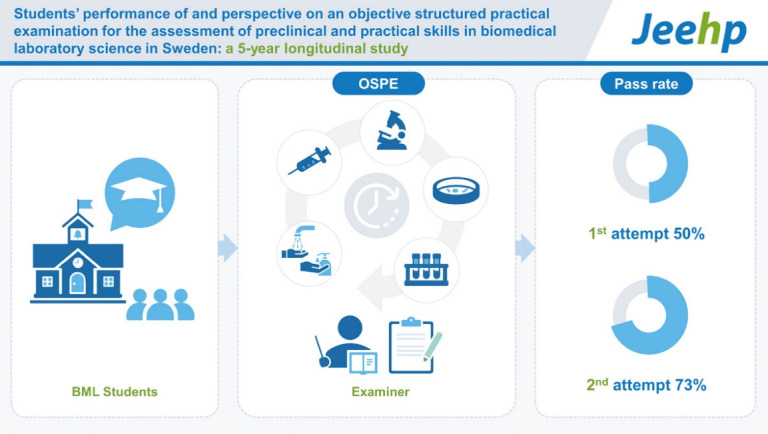


**Table 1.** Student characteristics, including gender and age at the participation of the different objective structured practical examination sessions

Year	Age (yr)	Women (%)	Men (%)
2015 (N=38)	24.4±4.76	31 (83.3)	7 (18.4)
2016 (N=47)	28.1±8.23	40 (85.1)	7 (14.9)
2017 (N=37)	27.7±7.02	26 (70.3)	11 (29.7)
2018 (N=31)	27.4±7.56	27(87.1)	4 (12.9)
2019 (N=42)	27.4±6.56	33 (78.6)	9 (21.4)
Total (N=195)	27.0±7.01	157 (80.5)	38 (19.5)

Values are presented as mean±standard variation or number (%).

## Footnotes

**Data availability**
Data files are available from Harvard Dataverse: https://doi.org/10.7910/DVN/JDK6GM Dataset 1. Raw data from OSPE by year.

Dataset 2. Students’ response to OSPE and survey questionnaire by year.

## Supplementary materials

Supplementary files are available from Harvard Dataverse: https://doi.org/10.7910/DVN/JDK6GM

Supplement 1. Implementation process of an objective structured practical examination.

Supplement 2. Detailed intended learning outcomes per objective structured practical examination station.

Supplement 3. Student survey questions.

Supplement 4. Semi-structured interview protocol for examiners.

Supplement 5. Audio recording of the abstract.
