# Supplementary material for: Students’ performance of and perspective on an objective structured practical examination for the assessment of preclinical and practical skills in biomedical laboratory science students in Sweden: a 5-year longitudinal study
Source: J Educ Eval Health Prof. 2023 Apr 6;20:13. doi: 10.3352/jeehp.2023.20.13 (PMC10175044; doi:10.3352/jeehp.2023.20.13)
Supplement: Supplementary file 3 — Supplement 1. Implementation process of an objective structured practical examination. [file jeehp-20-13-suppl1.docx]

Supplement 1. Implementation process of an objective structured practical examination

**Establishing the OSPE**

The objective structured practical examination (OSPE) was divided into ten different stations, for each station, there was a period of 12 minutes for the students to complete their task, and thereafter 3 minutes to rotate from one station to the next. The structure of the different stations was based on five different competencies linked to individual intended learning outcomes (ILOs), i.e., sampling, aseptic techniques, quality assurance, and general and laboratory skills, as outlined in figure 0. In some stations, all 5 different competencies were assessed in others only some of them. For each station, there was an examiner’s protocol with guidelines in case that the student had questions. In this protocol, it was stated what the examiner was allowed to answer and in which way. Each examiner graded individual student performance using a station checklist comprising a varying number of items per checklist based on the task assessed with 10 to 20 items per checklist. Each item of the checklist was scored according to the binary system, that is, the “yes/no” scale, by the examiner, and marks were given accordingly. Of the individual checklist items, 2 to 4 were global criteria. After completion of the entire OSPE, all checklists were reviewed and the individual stations were then classified as passed, borderline, or failed stations. A station was considered borderline if the student needed assistance to be able to pass a global criterion. To pass the OSPE all ILOs must be assessed as approved. If a student failed one or more of the ILOs, the OSPE was assessed as failed and the student had to rerun all failed or borderline stations including this ILO in a new OSPE setting. Two extra OSPE examinations were therefore performed to assess students who either were unable to participate or failed the original OSPE.
