# Supplementary material for: Students’ performance of and perspective on an objective structured practical examination for the assessment of preclinical and practical skills in biomedical laboratory science students in Sweden: a 5-year longitudinal study
Source: J Educ Eval Health Prof. 2023 Apr 6;20:13. doi: 10.3352/jeehp.2023.20.13 (PMC10175044; doi:10.3352/jeehp.2023.20.13)
Supplement: Supplementary file 5 — Supplement 3. Student survey questions. [file jeehp-20-13-suppl3.docx]

**Supplement 3.** Student survey questions

**Course evaluation**

Mark the score you find best agrees with your opinion. The levels are scored according to 1=I do not agree at all and 5=I agree. I assess that I have achieved all the learning objectives of the course.

**Question 1**

I have through this course developed valuable knowledge/skills.

**Question 2**

I assess that I have achieved all the learning objectives of the course.

**Question 3**

I perceive that there was an alignment within the course from learning objectives to examination.

**Question 4**

I perceive that the course has stimulated me to a scientific approach (for example, analytical and critical thinking, own search, and evaluation of information).

**Question 5**

I understand that the teachers were accommodating during the course for ideas and opinions on the design of the course.

**Question 6**

I felt that the course contributed to my being well-prepared for my future profession.

**Question 7**

The course built on my knowledge from previous courses during the education.

**Question 8**

I have received useful feedback.

**Question 9**

We, students, were encouraged to take responsibility for our learning.

**Question 10**

The course was research-related.

**Question 11**

Overall, I am satisfied with this course.
