# Supplementary material for: Students’ performance of and perspective on an objective structured practical examination for the assessment of preclinical and practical skills in biomedical laboratory science students in Sweden: a 5-year longitudinal study
Source: J Educ Eval Health Prof. 2023 Apr 6;20:13. doi: 10.3352/jeehp.2023.20.13 (PMC10175044; doi:10.3352/jeehp.2023.20.13)
Supplement: Supplementary file 6 — Supplement 4. Semi-structured interview protocol for examiners. [file jeehp-20-13-suppl4.docx]

**Supplement 4.** Semi-structured interview protocol for examiners

**Semi-structured interview questions for examiners participating in the objective structured practical examination (OSPE)**

**Question 1**

Concerning the station assessed:

Was the task linked to the intended learning outcome to be examined at the station?

Was the time set aside for that task adequate?

**Question 2**

Concerning the checklist for the above station:

As an examiner, would you like to change any of the assessment criteria to the station, if so what/which and in what way? (e.g., were they too many/too few/too unclear?)

**Question 3**

Concerning the assessment of the above station:

Do you feel that the examination enabled an objective assessment (e.g., if the student was sad/upset/stressed)

**Question 4**

Concerning the “guidelines to examiners” for the above station:

Are you missing something in the guidelines for the examiners and if so what?

**Question 5**

In relation to the individual students at the above station:

Did you experience the students as stressed, and if so in which way?

**Question 6**

Is there anything else you’d like to add concerning the OSPE?
